# Navon letters and composite faces: same or different processing mechanisms?

**DOI:** 10.3389/fpsyg.2023.1219821

**Published:** 2023-11-02

**Authors:** Daniel Fitousi, Omer Azizi

**Affiliations:** Department of Psychology, Ariel University, Ariel, Israel

**Keywords:** face recognition, Navon letters, LBA, response time models, holistic processing, composite face illusion

## Abstract

Navon letters and composite faces are two fascinating demonstrations of hierarchical organization in perception. Many researchers believe that the two types of stimuli and their associated tasks gauge comparable holistic mechanisms. This belief is so common that the two paradigms are now being applied in tandem to measure impaired holistic processing in prosopagnosic patients. But are Navon letters and composite faces processed in a similar fashion? In the present study we take a closer look at their apparent affinity. We gain novel insights into their underlying mechanisms by fitting parameters of the *linear ballistic accumulator* (LBA) model to empirical correct and incorrect response times (RTs). The results reveal major differences in processing between the two tasks. We conclude that despite the presence of a compelling surface similarity, Navon compound letters and composite faces tap into separate psychological processes.

## 1. Introduction

The Gestalt tradition in psychology (Wertheimer, 1912; Koffka, 1935) has exerted considerable influence on psychological thinking over the last hundred years or so (Wagemans et al., 2012; Algom and Fitousi, 2016). The main thrust of the Gestaltists' idea is that: “the whole is grasped even before the individual parts enter our consciousness… [and that] Gestalten are structures that are segregated from the background… to which the other parts are related hierarchically” (Wagemans et al., 2012, p.1175). Inspired by the Gestalt tradition, researchers have studied extensively holistic and hierarchical mechanisms. Particularly notable are studies in the fields of object recondition (Bar et al., 2006) and face perception (Farah et al., 1998; Fitousi, 2023). Two primary demonstrations of holistic processing are the composite face illusion (Galton, 1879; Young et al., 1987) and Navon's compound letters (Navon, 1977). Many researchers assume that composite faces and Navon compound letters are driven by comparable holistic mechanisms (Behrmann et al., 2005; Avidan et al., 2011). This assumption seems reasonable, but the evidence supporting this claim is not strong (Wang et al., 2012; Gerlach and Krumborg, 2014). The current study set to test this hypothesis directly. We asked whether Navon compound letters and composite faces are governed by the same psychological mechanisms. To address this question we have deployed the *linear ballistic accumulation model* (LBA, Brown and Heathcote, 2008)—a sequential sampling model of response times (RTs). In this approach, performance in each task is accounted for by a unique process model. This is accomplished by fitting the LBA's psychologically meaningful parameters to empirical correct RTs, incorrect RTs, and accuracy rates. To anticipate our results, we found major differences across the two tasks in model parameters, which in our view, reflect separate underlying mechanisms. These outcomes challenge the notion that composite faces and Navon figures are subjected to comparable processing mechanisms.

**Figure 1:**
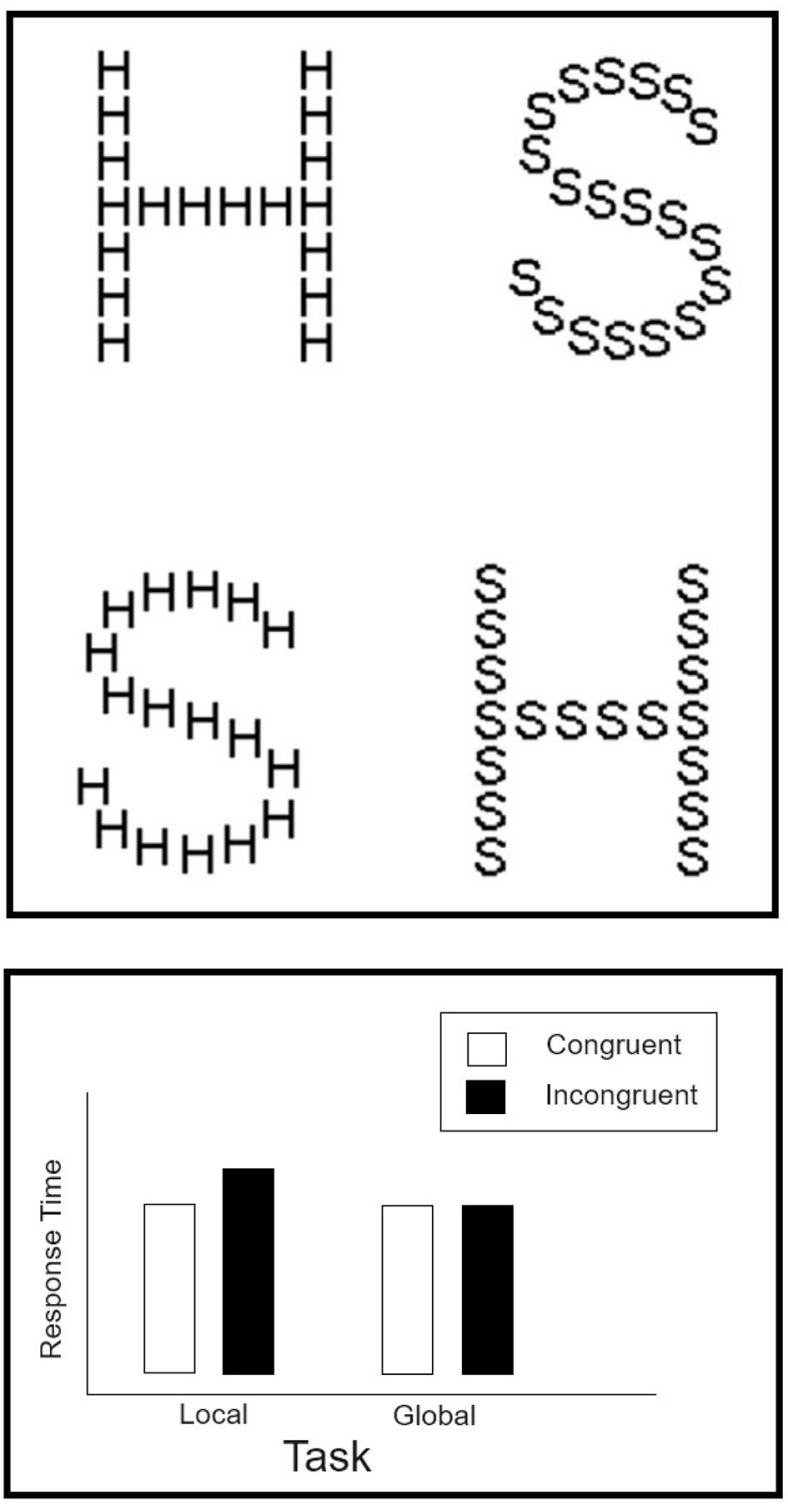
**(Top)** Examples of the Navon compound letters constructed from the English capital letters “H” and “S.” These stimuli were used in Experiment 1. **(Bottom)** Prototypical results from the Navon paradigm.

Navon figures and composite faces are classic examples of Gestalt phenomena. Both have been studied extensively over the last four decades or so, serving as strong markers of holistic processing. Navon compound letters (see Figure 1) are built hierarchically, by creating a large letter (“H”) that is composed of replicas of a physically small letter (“S”). Varying the identity of the global and local letters can lead to either *congruent* stimuli (e.g., large “H” composed of small “H”s) or *incongruent* stimuli (e.g., large “H” composed of small “S”s). When participants are asked to classify the local letter often a congruity effect emerges, such that participants are faster and more accurate in the congruent than in incongruent trials. A congruity effect is either absent or weaken when participants are asked to classify the identity of the global letter. This asymmetry has been interpreted as an evidence for the precedence of the global shape over constituent local parts (Navon, 1977). When attending to the local letter, people cannot ignore the meaning of the global letter—a failure of selective attention that leads to an interference. In contrast, when attending to the global letter, observers are not interfered by the local letters to the same extent (Kimchi, 1992). The asymmetry is a hallmark of the global precedence in vision (Navon, 1977).

**Figure 2:**
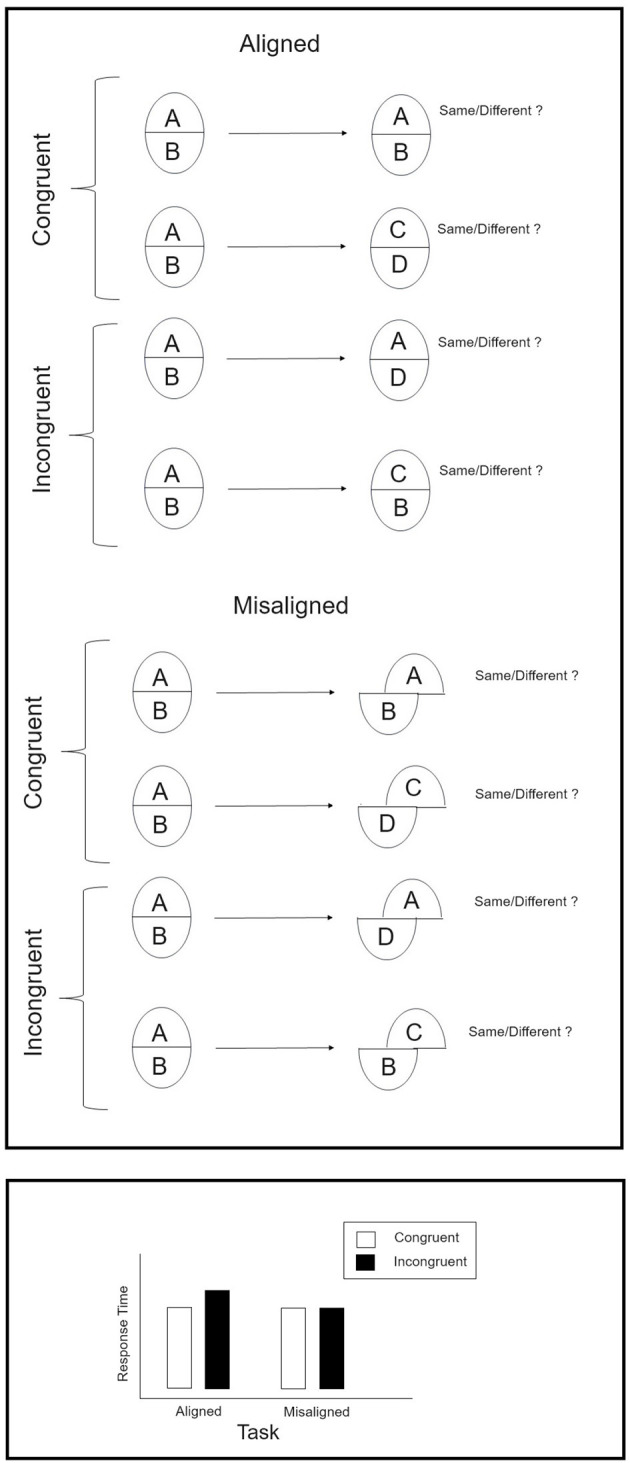
**(Top)** Schematic illustration of the “complete design” version of the composite face task. **(Bottom)** Prototypical results in this paradigm.

In the composite face illusion (Richler and Gauthier, 2014; Fitousi, 2015) two face halves from two different people are fused to create a third, new, and never-seen facial identity. The illusion is abolished when the face parts are misaligned, or when the face is inverted (Hole, 1994; Fitousi, 2020a). In the lab, the illusion is often measured by administrating the so called “complete design” (Richler and Gauthier, 2014). The top panel of Figure 2 provides a schematic illustration of this paradigm. On each trial participants are presented with a sequence of two composite faces—a “study” and a “test” composite face. Participants have to attend to the top-half of the test composite and indicate whether it is “same” or “different” from that of the study composite. The study and test composites can be either congruent (top is “same” and bottom is “same” or top is “different” and bottom is “different”), or incognruent (top is “same” but bottom is “different,” or top is “different” but bottom is “same”). The task is performed under two conditions. In the aligned condition, the two face halves are posited one above the other to provide the impression of a whole face. In the misaligned task, the top half is shifted with respect to the bottom half in order to breakdown the holistic nature of the face. Aligned and misaligned stimuli are often presented in separate blocks. The bottom panel of Figure 2 presents a set of prototypical results in this paradigm. For aligned faces, a congruity effect is observed such that response times (and accuracy) are better in congruent than in incogruent trials. This congruity effect is not observed for misaligned composites. The manner by which this illusion exerts its unique influence is still a matter of debate (Richler et al., 2012; Rossion, 2013). However, the consensus is that the illusion is the result of a holistic strategy, by which the constituent parts are subject to a global influence of the whole face (Behrmann et al., 2005; Avidan et al., 2011; Busigny and Rossion, 2011).

The similarity between Navon compound letters and composite faces is remarkable. First, both type of stimuli exert strong phenomenological impression of conflict between opposing sources of information. Second, both yield a statistical interaction between factors of Congruity and Task (the reader is invited to compare the bottom panels of Figures 1, 2). Third, researchers believe that both are subjected to comparable holistic processes by which the global or whole object (face) has some precedence over the local (part, feature) constituting elements (Busigny and Rossion, 2011). Faces are hierarchical objects in the same sense that Navon figures are. A classic definition of hierarchical objects is offered in the seminal paper by Kimchi (1992). She defines hierarchical objects by distinguishing between two levels of processing: “a visual object, viewed as a whole, has both wholistic properties and component properties/parts. Wholistic properties are properties that depend on the interrelation between the component parts (e.g., Navon, 1977; Garner, 1978; Rock, 1986)” (p. 25). Faces easily meet this definition. They are wholistic entities composed of properties/parts (e.g., eyes, nose). The whole is often perceived as the interrelations (second-order relations) between individual features (eyes, nose, mouth) (Tanaka and Farah, 1993). In the case of composite faces, the whole (global) level is generated by the alignment of top- and bottom-halves. This “wholeness” is disrupted by misaligning the parts. This allows participant good selective attention to the top-part without suffering interference from the bottom part. Similarly, good selective attention is afforded with Navon compound figures, when attention is directed to the global level. In earnest it should be noted that current definitions of “holistic” and “Gestalt” perception are fraught with inconsistency and ambiguity (Garner and Morton, 1969; Maurer et al., 2002; Richler et al., 2012; Fitousi, 2013). Here we adhere to the most common definition.

The tasks deployed in the composite face and Navon paradigms are relatively simple. In both tasks, the experimenter measures response times (RTs) and accuracy rates in a two-alternative choice decision. In both tasks, the degree to which the constituent elements create a good configuration is manipulated experimentally. In the composite face task, alteration of configurality is achieved via alignment and congruency of the top and bottom face parts. In the Navon task, configurality is manipulated via the alteration of the hierarchical structure of the global letter and local letters, and the relevant dimension to which the participant is asked to attend. For both tasks, researchers have elicited rival sensory (Miller and Navon, 2002; Andres and Fernandes, 2006; Rossion, 2013) and attentional (Shulman and Wilson, 1987; Richler and Gauthier, 2014) mechanisms, but with no apparent resolution on the winning theoretical account so far (Kimchi, 1992; Richler et al., 2008).

The Navon task is now routinely used in tandem with the composite face task as a means of assessing sundry aspects of holistic perception in prosopagnosic patients (Behrmann et al., 2005; Duchaine et al., 2007a,b; Avidan et al., 2011; Busigny and Rossion, 2011). The logic sustaining these studies is that deficits in face perception are due to impaired holistic processing, and thus these deficits extend to other stimuli requiring holistic processing, including the Navon figures. To a first approximation, the assumption that the two tasks engage the same holistic mechanisms seems plausible. However, the evidence in favor of this hypothesis is not particularly compelling (Wang et al., 2012; Gerlach and Krumborg, 2014). For example, Busigny and Rossion (2011) reported on a prosopagnosic patient who showed impaired composite face effect, but intact global letter interference. A similar result has been found by Duchaine et al. (2007a,b). If indeed, the two tasks reflect a common holistic mechanism, one should have observed impairment in both tasks. Furthermore, Gerlach and Krumborg (2014) have found considerable inconsistencies and low reliability in performance across- and within- participants and across types of Navon figures. As a result, they questioned the validity of studies relating Navon measures of global/local processing to face processing.

## 2. Moving beyond surface similarity of Navon figures and composite faces

The affinity between Navon letters and composite faces also extends to a common statistical signature on mean RTs and accuracy. Both tasks are measured as an interaction between Congruity and Task. This common statistical regularity has likely contributed to the view that the two tasks tap into the same process. But the question still remains. Is there a deeper common psychological mechanism that justifies their joint application? Note that taking the prototypical effects on mean RTs and accuracy at face value is problematic. The relations between accuracy and RTs can be complex. For example, incorrect RTs can be faster than correct RTs in one task, but slower than correct RTs in the other. Traditional mean RTs analyses cannot readily account for such patterns. Moreover, speed-accuracy tradeoffs are also frequent patterns that call for a formal treatment (Ratcliff and Rouder, 1998). Such a formal treatment is afforded by the *Linear Ballistic Accumulator model* (LBA, Brown and Heathcote, 2008). Our main hypothesis is that if composite faces and Navon letters are governed by the same psychological mechanism/s they should exhibit the comparable effects on the parameters of the LBA.

## 3. The current study: applying the LBA to Navon letters and composite faces

Sequential sampling models (Ratcliff, 1978; Smith and Vickers, 1988; Busemeyer and Townsend, 1993; Usher and McClelland, 2001) can account for both speed and accuracy by deploying a relatively small number of decision-process variables. The models may differ with respect to the exact details of the underlying processes, but they do share some basic principles. These models assume that when an observer makes a decision in a speeded forced-choice task, he or she is sampling repeatedly from the stimulus, and this sampled evidence is accumulated in favor of one of the two responses. When the evidence for one of the responses reaches a bound, the decision process ends, and response is emitted. The time that is needed to emit the response is equal to the sum of decision time + the time that is needed for non-decision processes (e.g., stimulus encoding, response preparation). Sequential sampling models allow researchers to estimate three key variables: *drift rate*, which captures the rate by which evidence is accumulated, *response threshold* which captures how much evidence is needed before a decision is made, and *non-decision time*, which gives the estimated time of such operations as stimulus encoding and motor execution. These main parameters (along with a few others) offer a complete process model of the task, and can account for complex patterns of RTs and accuracy. They can explain entire RT distributions and speed-accuracy relations that simple ANOVAs, for example, cannot.

**Figure 3:**
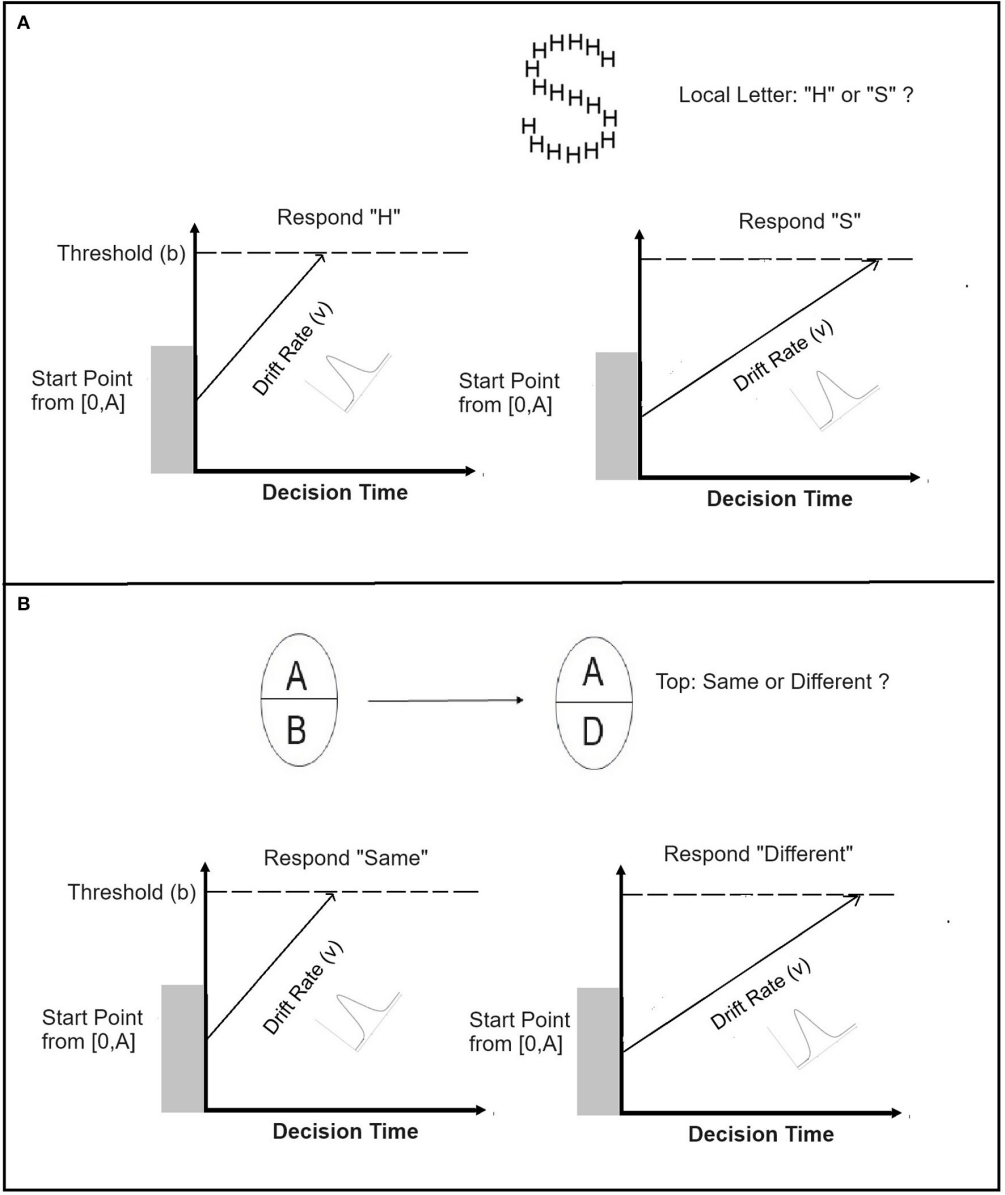
Application of the linear ballistic accumulator model (LBA) to decision in the Navon **(A)** and Composite face **(B)** tasks. Top panel: Two LBA accumulators for correct and incorrect responses, respectively, in the Navon task. Participants judge the local letter and ignore the global letter. Bottom panel: Two LBA accumulators for correct and incorrect responses, respectively, in the composite face task. Participants judge the top-half while ignoring the bottom-half.

Figure 3 illustrates the decision process in a pair of LBA accumulators. Suppose that these accumulators represent accumulation of evidence for the correct and incorrect responses. In the Navon task (see Figure 3A), participants classify the local (global) letter as either “H” or “S” while ignoring the identity of the local (global) letter. In the composite task (see Figure 3B), participants judge whether the top half of a test face is “same” or “different” from the top half of a study face while ignoring the status of the bottom-half. This decision is a two-alternative choice. The presentation of the stimulus leads to accumulation of evidence for both correct and incorrect responses, separately in the two corresponding accumulators (see Figure 3). The amount of evidence that is being accumulated is represented by the vertical axis, while the decision time is represented by the horizontal axis. Evidence increases linearly. The slopes of the lines reflect the rate at which the evidence increases in each accumulator. The *drift rate* is determined by the parameter *v*. In LBA there is a drift rate for each accumulator. The relative magnitude of the drift rate parameter captures differences in task performance across conditions. Drift rates in LBA vary randomly from trial-to-trial independently for each accumulator according a normal distribution with mean *v* and standard deviation *s*. This variability captures fluctuation in random factors such as attention or internal noise. The starting point A represents the amount of evidence in each accumulator before accumulation of evidence begins. The starting point of each accumulator varies from trial-to-trial according to a uniform distribution [0,A]. The response threshold is determined by parameter *b*, which is constrained by the value of A, meaning that a response can be made after accumulating some evidence. Speed-accuracy trade-offs in which the preferred response is faster, emitted more often, but is less accurate, can be accounted for by assuming that the thresholds in the two accumulators are identical, but observers favor one response over the other by reducing the value of the starting point *b* of the corresponding accumulator. The time it takes for accumulated evidence to reach threshold on a given trial equals the distance between the response threshold and the start point, divided by the rate of evidence accumulation (Brown and Heathcote, 2008). The total RT in a trial equals the sum of decision time and non-decision time *Ter*, which is also an important parameter in the model. The LBA parameters are fitted to the data using closed form formulas (Brown and Heathcote, 2008).

A marked advantage of LBA (and other sequential sampling models) over extant approaches to RT analyses is that it offers a complete process model of the underlying psychological components (Donkin et al., 2009), one that takes into account both RT distributions and accuracy rates. The LBA parameters are amenable to viable psychological interpretations. These parameters are known to be *selectively influenced* by well-defined psychological variables (Donkin et al., 2011; Fitousi, 2020b). For example, the drift-rate parameter *v* is affected by such factors as stimulus quality and task difficulty, whereas the threshold parameter *b* is sensitive to changes in response caution. The non-decision *Ter* is affected by psychological variables associated with encoding or response execution (Voss et al., 2004; Donkin et al., 2011). Fitting the LBA to RT and accuracy data from the composite-face and Navon tasks will allow us to: (a) provide a rigorous process model for each task, (b) identify the LBA parameters that generate the composite face effect and the Navon effect, (c) compare the two phenomena with respect to these parameters, and lastly (d) offer psychological interpretations (Donkin et al., 2011; Fitousi, 2020b) of these differences in processing. To the best of our knowledge this is the first time that the LBA (or any other sequential sampling model) is applied to the composite face and Navon tasks. We decided to administer the composite face task and the Navon task with two independent groups of participants. There is evidence (Gerlach and Krumborg, 2014) that tasks that induce holistic mode of processing, such as the Navon task, can carry over to subsequent tasks (such as the composite face task). Thus, in order to avoid these unwarranted interactions, and to record pure measures of performance in each task, we fit the LBA to data from two independent groups of participants that performed these tasks. In Experiment 1 we fit the LBA to data from a new experiment with the Navon compound letters. In Experiment 2, we fit the LBA to a data from a composite face study by Fitousi (2019).

LBA analyses provide us with well-defined quantitative measures of concrete psychological mechanisms, whereas current verbal definitions of “holism” are fraught with ambiguity and inconsistency and are not amenable to explicit quantitative and psychological interpretation. The LBA allows us to distinguish between perceptual and other (e.g., decisional) sources of hierarchical phenomena. The former should be reflected as changes in drift-rates, whereas that latter (e.g., motor, encoding, or post-perceptual) should manifest as changes to non-decision times and response threshold parameters. We hypothesized that if hierarchical objects and faces are governed by a common psychological mechanism, they should exhibit comparable effects on the same set of LBA parameters.

## 4. Experiment 1

In this Experiment participants performed in the Navon's letter task. The goals of this experiment were threefold: (a) replicate the global precedence phenomenon captured by Navon's letters, (b) fit LBA models to RT and accuracy, and (c) obtain the parameter values for their psychological interpretation and then (in Experiment 2) compare them with those obtained for the composite face illusion.

## 5. Methods

### 5.1. Participants

Thirty six participants from Ariel University (mean age = 24.47, sd = 3.82, Females = 23, Males = 13), were recruited. They received course credit for their participation. All reported normal or corrected-to-normal vision. All gave their approved consent.

### 5.2. Stimuli and apparatus

The stimuli consisted of four Navon figures created with the capital letters “H” and “S.” The Navon figures were presented as black characters over white background. The height of the global letters was 1.5 cm and its width 0.8 cm. Viewed from a distance of 56 cm, each global letter subtended 0.026° of visual angle vertically and 0.014° horizontally. Each global letter was comprised of 18 local letters. The height of each local letter was approximately 0.02 cm and its width 0.01 cm. Thus, each local letter subtended 0.00035° of visual angle vertically and 0.00017° horizontally. The Navon figures consisted of four stimuli. Two congruent figures: a large “H” comprised of small “H”s and a large “S” comprised of small “S”s; and two incongruent figures: a large “H” made out of small “S”s, and a large “S” constructed from small “H”s (see Figure 1). On each trial, one of these four Navon figures was presented at the center of the screen. The exact location of the letter was subjected to spatial uncertainty of up to 10 pixels on the vertical and horizontal dimensions. Spatial uncertainty was introduced to eliminate the possibility that observers will know in advance the exact location of the stimuli and will be able to adopt a focusing strategy.

### 5.3. Design and procedure

Participants performed in two separate tasks. In the local-letter task, they categorized the small letters as either “H” or “S,” while ignoring the identity of the large letter. In the global-letter task, participants classified the large letter as either “H” or “S,” while ignoring the identity of the small letters. Participants indicated their decisions by pressing a left or right key on the computer keyboard according to a specified key assignment. The order of the tasks was chosen randomly by the computer. Each block consisted of 48 trials. Participants completed 7 experimental blocks for each task (global, local). The four Navon letters could appear equally often. Overall, participants completed 672 trials. No feedback was provided. Instructions highlighted both speed and accuracy. A 1 min break was given after each block of trials. Participants were asked to be as accurate and as rapid as possible.

## 6. Results

### 6.1. RT and accuracy

**Figure 4:**
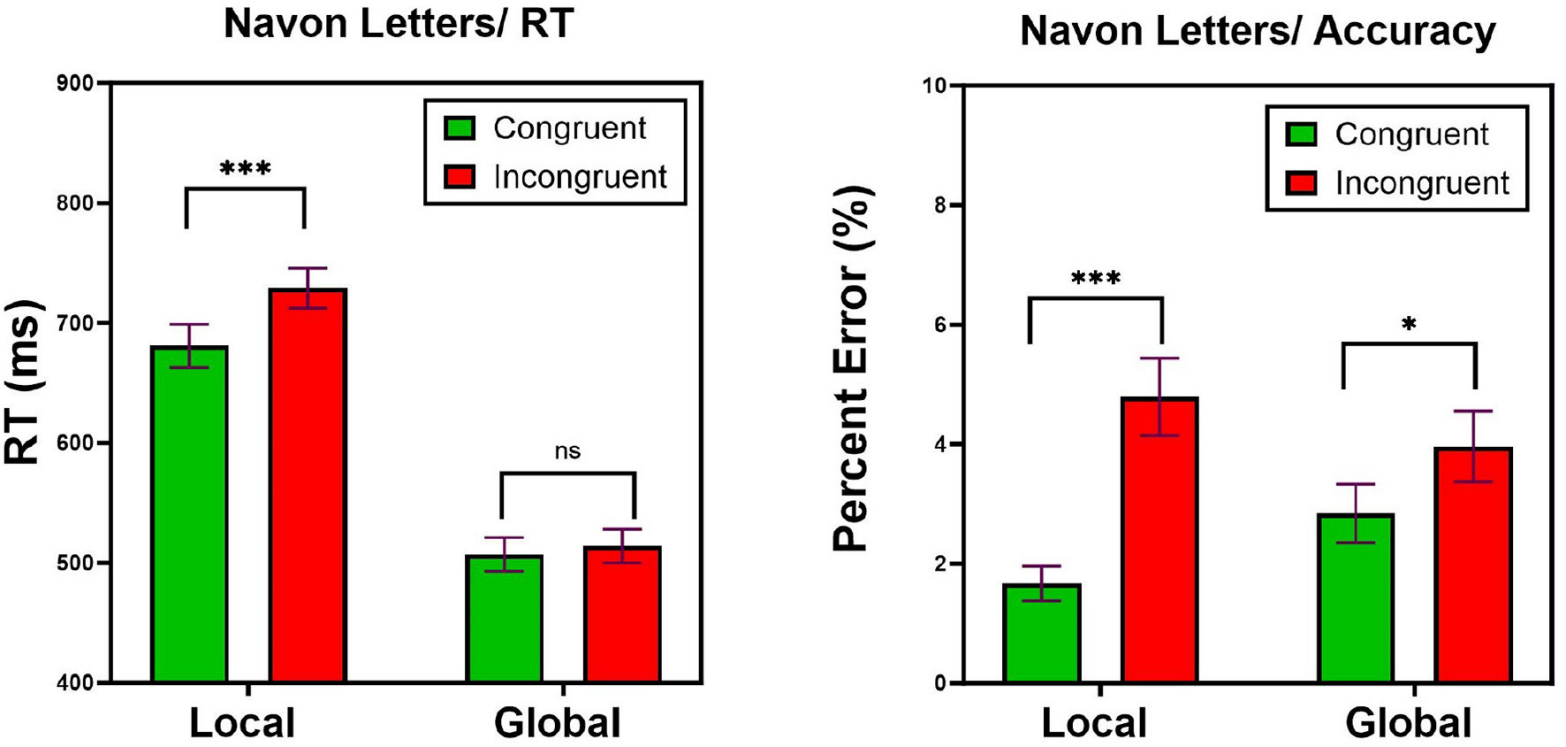
Experiment 1: Mean correct RTs **(left)** and Percentage of Error **(right)** as a function of Congruity (congruent, incongruent) and Task (local, global). Error bars are standard errors of the mean. n.s, non significant; ^*^p < 0.05, ^**^p < 0.01, ^***^p < 0.001.

Trials with RT shorter than 100 ms, longer than 2,500 ms (0%), or incorrect trials (3.3%) were removed from analyses. Note that the incorrect trials were not removed from the LBA analyses because in this model they serve as a major source of information. One participant committed high rate of errors in the global-letter task (44%) and their data were not included in the analyses. Mean incorrect RTs (607 ms) was not different from mean correct RTs (593 ms) [*t* < 1]. Mean RTs for correct responses, as well as percentage of error rates are presented in Figure 4. Visual inspection of this Figure suggests that the classic Navon pattern is replicated here. First, judgments of the global letter are more efficient than classification of the local letters. Second, the interference of the global letter with the local letter is much greater than the interference of the small letter with the larger letter. These patterns are supported by statistical analyses. A two-way ANOVA with Task (local, global) and Congruity (congruent, incongruent) revealed a main effect of Task [*F*_(1, 34)_ = 401.3, *MSE* = 1, 322, 660, *p* < 0.0001], entailing faster RTs in the global than in the local task. A main effect of congruity [*F*_(1, 34)_ = 57.18, *MSE* = 26, 886, *p* < 0.0001], pointed to faster RTs in congruent than incongruent trials. Most importantly, the Task x Congruity interaction was significant [*F*_(1, 34)_ = 31.77, *MSE* = 15, 106, *p* < 0.0001], supporting the asymmetric pattern by which the global dimension interfered (48 msec) with the local dimension [*F*_(1, 34)_ = 53.95, *MSE* = 41, 149, *p* < 0.0001] to a greater extent than did the local dimension with the global dimension (7 msec) [*F*_(1, 34)_ = 4.60, *MSE* = 843.1, *p* < 0.05]. Comparable analyses on error rates revealed no effect of task [*F* < 1], but did show a main effect of Congruity [*F*_(1, 34)_ = 37.56, *MSE* = 0.015, *p* < 0.0001], and most importantly, a significant Task x Congruity interaction [*F*_(1, 34)_ = 15.45, *MSE* = 0.003, *p* < 0.001] that mimicked the asymmetric pattern observed with RTs, namely a larger interference (3.22%) from the global to the local dimension [*F*_(1, 34)_ = 36.56, *MSE* = 0.017, *p* < 0.0001] than from the local to the global dimension (1.12%) [*F*_(1, 34)_ = 12.19, *MSE* = 0.0021, *p* < 0.001]. Taken together, these results document the processing advantage of the global dimension over the local dimension (Navon, 1977), and the breakdown of selective attention to the local letter. Now, the question is what psychological mechanisms can account for these patterns? The next LBA analysis can provide the answer.

### 6.2. LBA analyses

**Figure 5:**
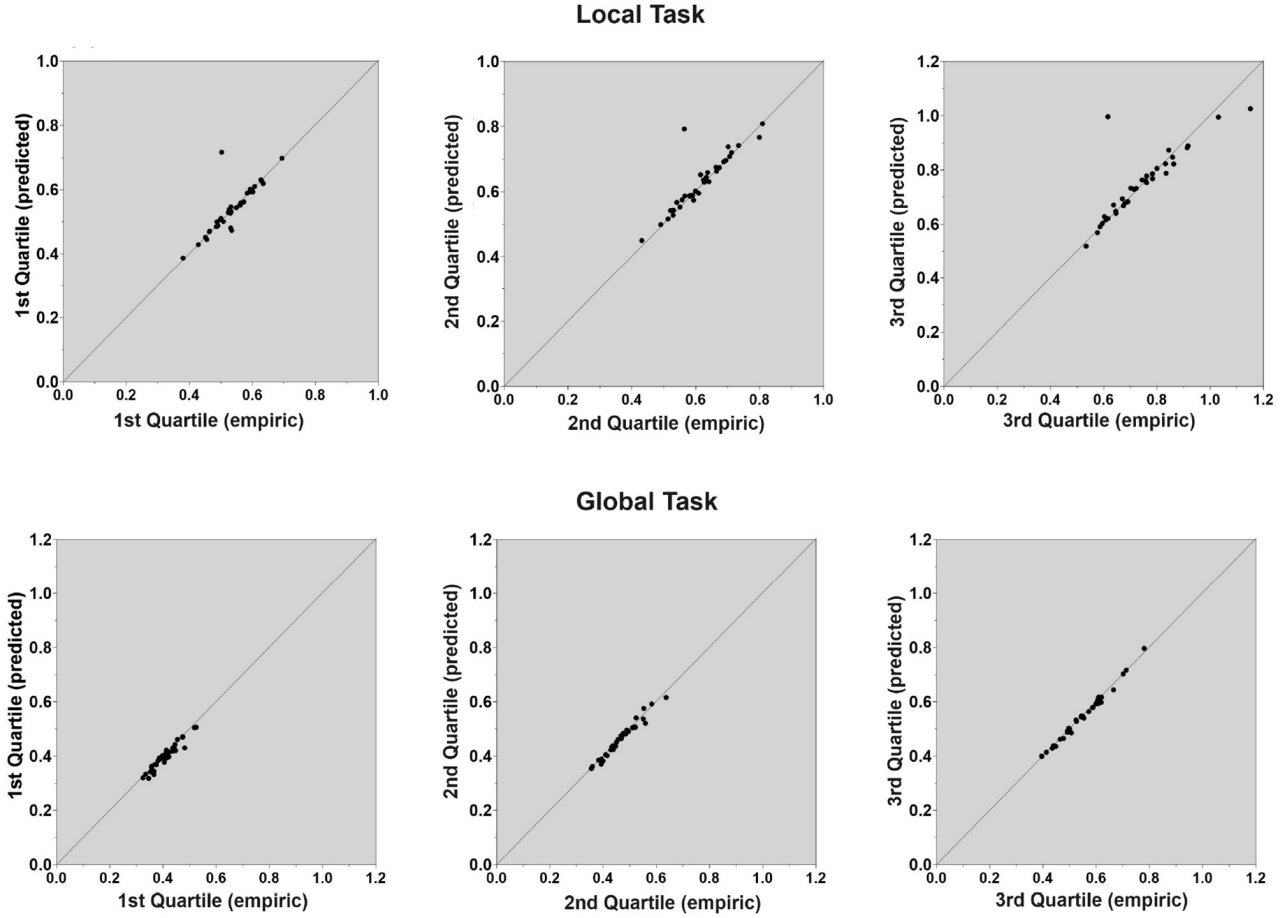
Experiment 1: Quality of fit of the LBA model parameters to the data in judgments of the local letter **(top)** and in judgments of the global letter **(bottom)**. Quartile-Quartile plots are depicted for the 1st, 2nd, and 3rd Quartiles in which empirical mean RTs (in seconds) are plotted against predicted mean RTs (in seconds).

The LBA is comprised of five parameters (*b*, A, *s, Ter, v*) that determine correct and incorrect RTs as well as the accuracy rate in a given experimental condition. When modeling simultaneously a number of experimental conditions, the number of parameters can be relatively high. For example, in the current study there are four experimental conditions [Congruity (2) × Task (2)], which require more than 40 parameters (5 LBA parameters × 4 conditions × 2 accumulators). The number of free parameters therefore should be constrained (Donkin et al., 2009; Fitousi, 2020b) by keeping some parameters constant across conditions, while allowing the most theoretically plausible parameters to vary. Thus, we fit the LBA for each task (global, local) separately and assume that RTs to congruent and incongruent trials are generated by different drift rates parameter and different non-decision time parameters, while the remaining three parameters (i.e., *s*, *b*, and *A*) are common to both levels of congruity. In other words, for each task we allowed the *drift-rate* and *non-decision time* parameters to vary freely across congruity levels, while keeping the *threshold* (b), *starting point*, (A) and standard variability of the drift rate (*s*) parameters the same within each congruity condition. The latter parameters did vary across blocks of tasks. This is an accordance with the view (Donkin et al., 2011) that participants do not change their decision criteria within but only across blocks. In our case, the tasks (i.e., global and local judgments) are blocked. We also made the plausible assumption that the accumulator parameters for the competing responses on a trial are the same, except for the drift rates, which we assumed to sum up to 1 (*v*_1_+*v*_2_ = 1). This assumption has been widely made in LBA applications (Brown and Heathcote, 2008; Donkin et al., 2011; Fitousi, 2020b). It implies that evidence for one response alternative reduces the evidence for the other response. Fitting was accomplished via the quantile maximum products estimation (QMPE, Heathcote et al., 2002), which takes into consideration both RTs and accuracy rates. Fitting was performed separately for each participant, with the goal of finding the best-fitting parameters that accounted for the participant's data. Then statistical inferences were made based on a set of ANOVAs on these values (Donkin et al., 2011). Figure 5 presents Quartile-Quartile plots in which the predicted mean RTs (in seconds) are plotted against the observed mean RTs (in seconds) for the first, second and third quartiles. As can be noted, quality of fit was very good with a slight advantage for the global task (Figure 5 Bottom) over the local (Figure 5 Top) task.

#### 6.2.1. Drift rates

**Figure 6:**
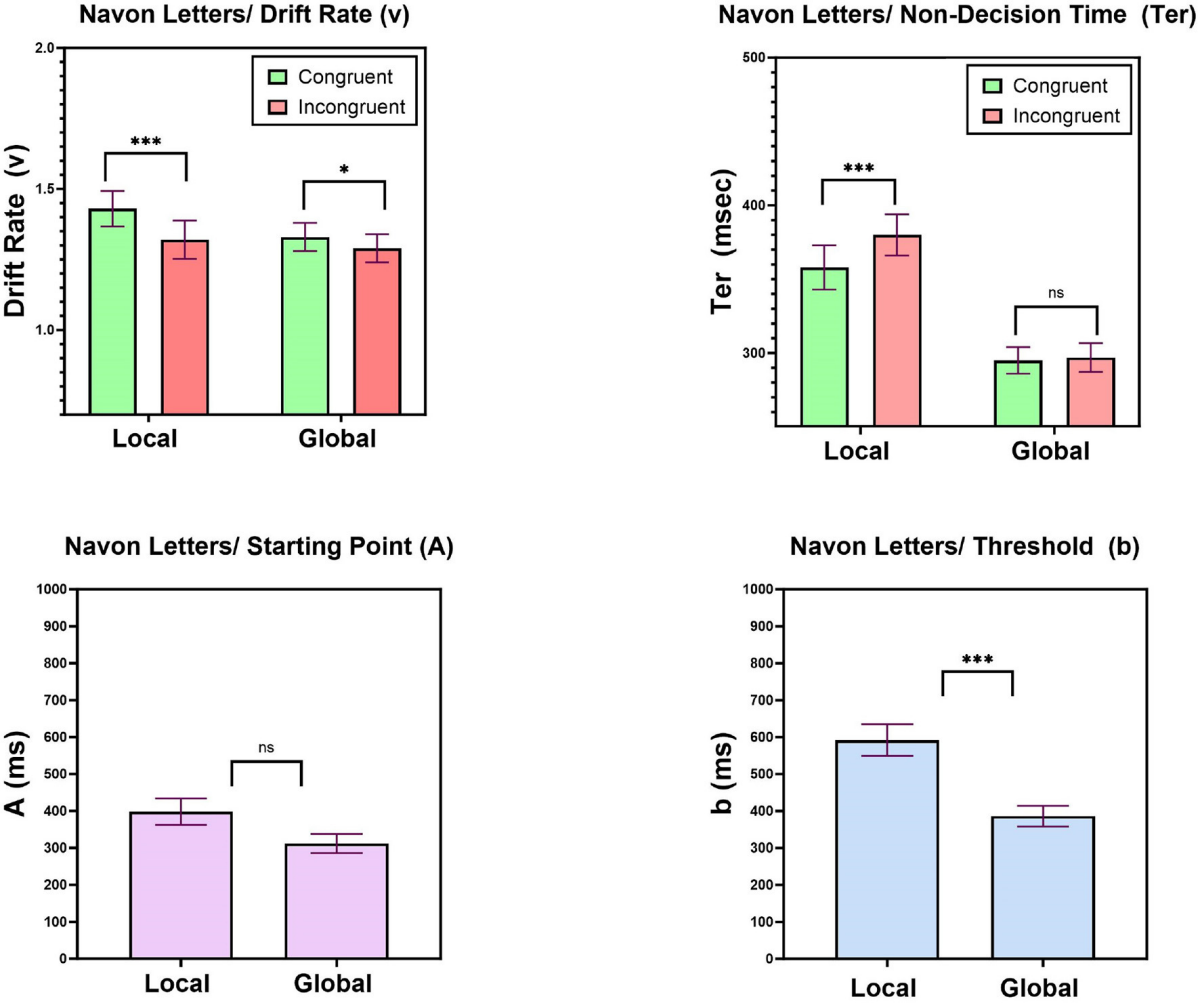
Experiment 1: Mean LBA parameters. **Top left**: drift rates (v) as a function of Task and Congruity, **Top right**: non-decision times (Ter) as a function of Task and Congruity. **Bottom left**: starting point (A) parameter as a function of Task (local, global); **Bottom right**: threshold (b) parameter as a function of Task (local, global). Error bars are standard error of the mean. n.s, non significant; ^*^p < 0.05, ^**^p < 0.01, ^***^p < 0.001.

Figure 6 top left presents mean values of the drift parameter (v) as a function of Congruity and Task. A two-way ANOVA with Task (local, global) and Congruity (congruent, incongruent) was conducted on the best-fitting drift rates. This analysis revealed that there was not effect of Task on drift rates [*F*_(1, 34)_ = 1.12, *MSE* = 0.14, *p* = 0.29], but did show a main effect of Congruity [*F*_(1, 34)_ = 18.16, *MSE* = 0.19, *p* < 0.001], such that higher drift-rates are recorded in congruent compared to incongruent trials. Most importantly, this Congruity effect was modulated by Task [*F*_(1, 34)_ = 4.43, *MSE* = 0.03, *p* < 0.05], such that a larger congruity effect obtained in judgments of the local letter [*F*_(1, 34)_ = 15.53, *MSE* = 0.20, *p* < 0.0001] than in classification of the global letter [*F*_(1, 34)_ = 4.99, *MSE* = 0.03, *p* < 0.05]. These results suggest that the congruity effect in the local task is partially derived by changes in the efficiency by which information is accumulated. In addition, the global and local tasks did not differ in terms of their drift rates.

#### 6.2.2. Non-decision time

Figure 6 top right gives mean values of the non-decision time parameter as a function of Congruity and Task. A two-way ANOVA with Task (local, global) and Congruity (congruent, incongruent) was performed on the best-fitting non-decision time (Ter) parameter values. A significant effect of Task [*F*_(1, 34)_ = 22.06, *MSE* = 188, 857, *p* < 0.0001], pointed to longer non-decision times in the local letter task (370 ms) compared to the global letter task (296 ms). In addition, a significant effect of Congruity [*F*_(1, 34)_ = 8.53, *MSE* = 5, 162, *p* < 0.01] was found which was modulated by Task [*F*_(1, 34)_ = 7.88, *MSE* = 3446, *p* < 0.01]. This interaction supported the presence of an asymmetric pattern by which the non-decision times in judgments of the local letter were 22 msec shorter in congruent compared to incongruent trials [*F*_(1, 34)_ = 9.51, *MSE* = 8, 522, *p* < 0.01], while a congruity effect was absent in classification of the global letter [*F* < 1]. These results give currency to the view that the global precedence with Navon figures can be ascribed, at least partially, to (a) shorter non-decision times in the global compared to the local task, and (b) shorter non-decision times in congruent compared to incongruet trials in the local task.

#### 6.2.3. Starting point (A) and threshold (b)

Recall that these parameters varied across tasks, but were held equal for congruent and incongruent trials within a given task. The rational was that participants do not alter their decision criteria within a block of trials, but only across blocks. The central question with respect to these parameters is therefore whether they differ across the global-letter and local-letter tasks. To answer this question, we compared their best-fitting values across tasks. Figure 6 bottom left, right present the mean LBA parameters of threshold (b) and starting point (b) as a function of task (local, global). For the starting point parameter (A), we did not find a difference between their value in local (398 ms, SE = 36.6 ms) and global (312 ms, SE = 26.5) tasks [*t*_(34)_ = 1.89, *p* = 0.067], whereas for the threshold parameter (b), we did observe a significant difference [*t*_(34)_ = 7.90, *p* < 0.0001], with higher threshold in local-letter task (592 ms SE = 43.2) compared to global-letter task (386 ms, SE = 28.73). These results suggest that one of the contributing psychological mechanism responsible for the global precedence is the alteration of response threshold (b) in the local letter task. It is likely that participants become more conservative in their decision when judging the local dimension.

## 7. Discussion

Experiment 1 replicated the global precedence phenomenon with Navon compound letters for both correct mean RTs and accuracy rates. Participants were more efficient at processing global letters than local letters, and most importantly, participants were interfered by the global letter to a larger extant than by the local letter. We fit LBA model parameters to RT distributions and accuracy rates from this experiment and found three main results. First, the asymmetric pattern was reflected in both the drift rate and non-decision parameters. Higher drift rate was observed in congruent than incongruent trials (i.e., a congruency effect), and this congruity effect was larger in local than global-letter task. Similarly, non-decision time was shorter in congruent compared to incongruent trials, and this congruity effect was found only in judgments of local-letters. Second, the more efficient performance in the global compared to the local task was reflected in the effects of non-decision (shorter in global compared to local task) and in threshold (lower in global compared to local task), with no influence of drift rate and starting point. Taken together, these results suggests that the Navon phenomenon is driven by increase in drift rate and decrease in non-decision times for congruent trials in the local letter task, and an increase in threshold under the local letter task. These point to both perceptual (drift rate), encoding and execution (non-decision times), and decisional (threshold) mechanisms that generate the Navon compound letter phenomenon. In the next experiment, we will derive similar LBA parameters for the composite face effect, and compare the underlying mechanisms with those of the Navon letters.

## 8. Experiment 2

The data for this experiment were collected in a study by Fitousi (2019). In his Experiment 1 the composite face effect was documented with both correct RTs and accuracy rates. Here we fit the LBA to the data from this Experiment. We provide a brief description of the experiment, while a complete and more detailed description can be found in the original paper. On each trial, participants were presented with a study composite face and then by a test composite face (see Figure 2). The task was to indicate whether the top half of the test composite face is “same” or “different” from the top half of the study composite face. Participants responded by pressing one of two response keys. Top and bottom parts varied orthogonally across trials, a manipulation which resulted in congruent and incongruent trials. In congruent trials, top part and bottom parts required compatible responses (e.g., both “different” or both “same”). In incongruent trials, top and bottom halves required incompatible responses (top is “same” and bottom is “different,” or top is “different” and bottom is “same”). In addition, the parts comprising the test face could be either aligned or misaligned (see Figure 2). The alignment manipulation was held across blocks. The two critical variables were Congruity and Alignment. This popular paradigm that gauges the composite face illusion is dubbed the “complete design” (Richler and Gauthier, 2014; Fitousi, 2019). It captures a Congruity × Alignment interaction by which participants exhibit better performance in congruent than incongruent trials only when the face parts are aligned. This result arguably captures the holistic or configural nature of face recognition (Richler and Gauthier, 2014). The question of interest concerns the psychological mechanisms that govern the this interaction. The LBA affords us to model both RTs and accuracy rates to uncover the exact psychological components (e.g., drift rates, thresholds, non decision times) responsible for this allegedly holistic pattern and compare them to those of the Navon letters.

## 9. Results

### 9.1. RT and accuracy

**Figure 7:**
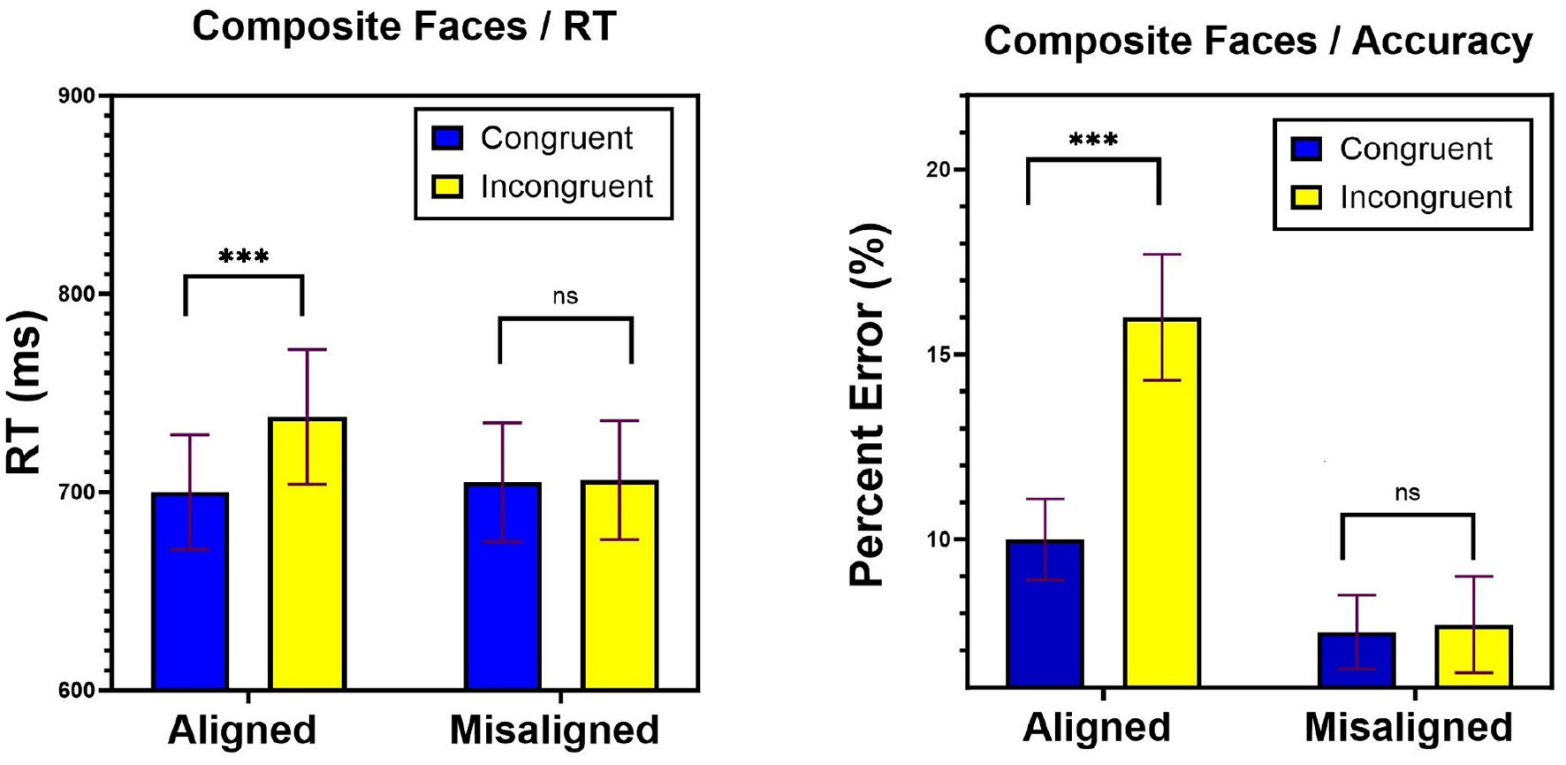
Experiment 2: mean correct RTs **(left)** and percentage of error **(right)** as a function of Task (aligned, misaligned) and Congruity (congruent, incongruent) conditions. Error bars are standard errors of the mean. n.s, non significant; ^*^p < 0.05, ^**^p < 0.01, ^***^p < 0.001.

Trials with RT shorter than 100 ms, longer than 2,500 ms (1%), or incorrect trials (10.7%) were excluded from analyses. Note that the incorrect trials were not removed in the LBA analyses because in this model they serve as a major source of information. Overall, incorrect RTs (847 ms) were slower than correct RTs (712 ms) [*t*_(20)_ = −5.12, *p* < 0.0001]. This often occurs when the task is difficult, or when accuracy rather than speed are highlighted (Luce, 1986; Ratcliff and Rouder, 1998). In the current case, both accuracy and speed were highlighted, but the task was relatively difficult. While traditional approaches to RTs cannot account for this observation, the interplay between the LBA parameters can account for this results. The left panel of Figure 7 presents mean RTs for correct responses in the aligned and misaligned conditions. As can be noted, the pattern underscores a sizable congruity effect in the aligned condition but not in the misaligned task. This pattern is supported by statistical analyses. A two-way ANOVA with Task (aligned, misaligned) and Congruity (congruent, incongruent) was conducted. The effect of Task was not significant [*F*_(1, 20)_ = 2.29, *MSE* = 3, 913, *p* = 0.14]. The effect of Congruity [*F*_(1, 20)_ = 16.69, *MSE* = 7, 413, *p* < 0.001] was significant and modulated by task [*F*_(1, 20)_ = 12.58, *MSE* = 7, 232, *p* < 0.01]. A 38 msec interference of the bottom-part intruding on judgments of the top-part was observed in the aligned condition [*F*_(1, 20)_ = 16.78, *MSE* = 14, 645, *p* < 0.01], but not in the misaligned condition [*F* < 1]. This outcome replicates the classic composite face effect.

Comparable analyses on error rates were performed. Figure 7 right panel presents mean percentage of error rates in the aligned and misaligned conditions. The main effect of Task (aligned, misaligned) was significant *F*_(1, 20)_ = 72.31, *MSE* = 0.067, *p* < 0.0001], indicating higher error rates in aligned (13.3%) than in misaligned (7.6%) condition. This effect is consistent with the idea of global (or holistic) precedence. In addition, a main effect of Congruity [*F*_(1, 20)_ = 10.17, *MSE* = 0.019, *p* < 0.005], and most importantly, a significant Task × Congruity interaction [*F*_(1, 20)_ = 122.16, *MSE* = 0.017, *p* < 0.001] obtained that reflected the asymmetric pattern observed with RTs. These results strongly suggest that the composite bottom-part intruded on judgments of the top-part (5.93%) in the aligned condition [*F*_(1, 20)_ = 18.55, *MSE* = 0.036, *p* < 0.0001], but not in the misaligned condition [*F* < 1]. In combination, these results record the presence of a genuine composite face effect for both correct RTs and accuracy rates. Notably, this interaction bears similarity to that observed with Navon figures. We next asked what are the psychological mechanisms that allow for this pattern to emerge and whether they are the same as those underlying the Navon figures by applying the LBA model.

### 9.2. LBA analyses

**Figure 8:**
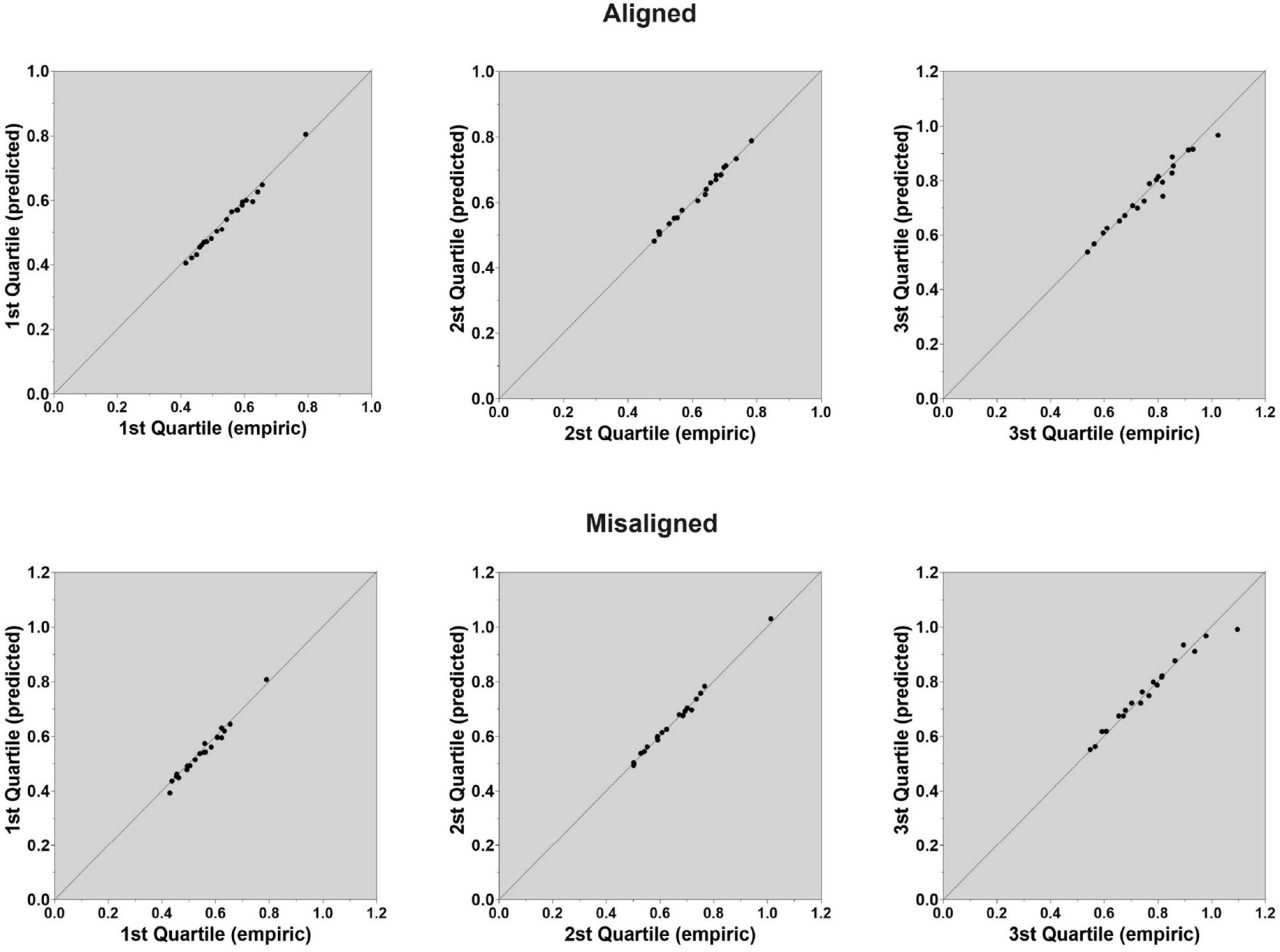
Experiment 2: Quality of fit of the LBA model parameters to the data in judgments of aligned composite faces **(top)** and in judgments of misaligned composite faces **(bottom)**. Quartile-Quartile plots are depicted for the 1st, 2nd, and 3rd Quartiles in which empirical mean RTs (in seconds) are plotted against predicted mean RTs (in seconds).

We fit the LBA parameters to the composite face data harnessing the exact same fitting routines as in Experiment 1. We assumed that participants did not alter their decision criteria within blocks of tasks, but only across blocks (Donkin et al., 2011). Thus, we fit the LBA parameters separately for the aligned and misaligned tasks and allowed *drift-rate* and *non-decision time* parameters to vary freely across congruity levels within a task, while holding the *threshold* (b), *starting point*, (A) and standard variability of the drift rate (*s*) parameters fixed within a task. The fitting algorithm converged for all participants. Figure 8 presents the observed mean RTs (in seconds) against the predicted mean RTs (in seconds) for each quartile. As can be noted, quality of fit was excellent. After deriving the best-fitting parameters for each participants in each task, we conducted a set of ANOVAs on these parameters values.

#### 9.2.1. Drift rates

**Figure 9:**
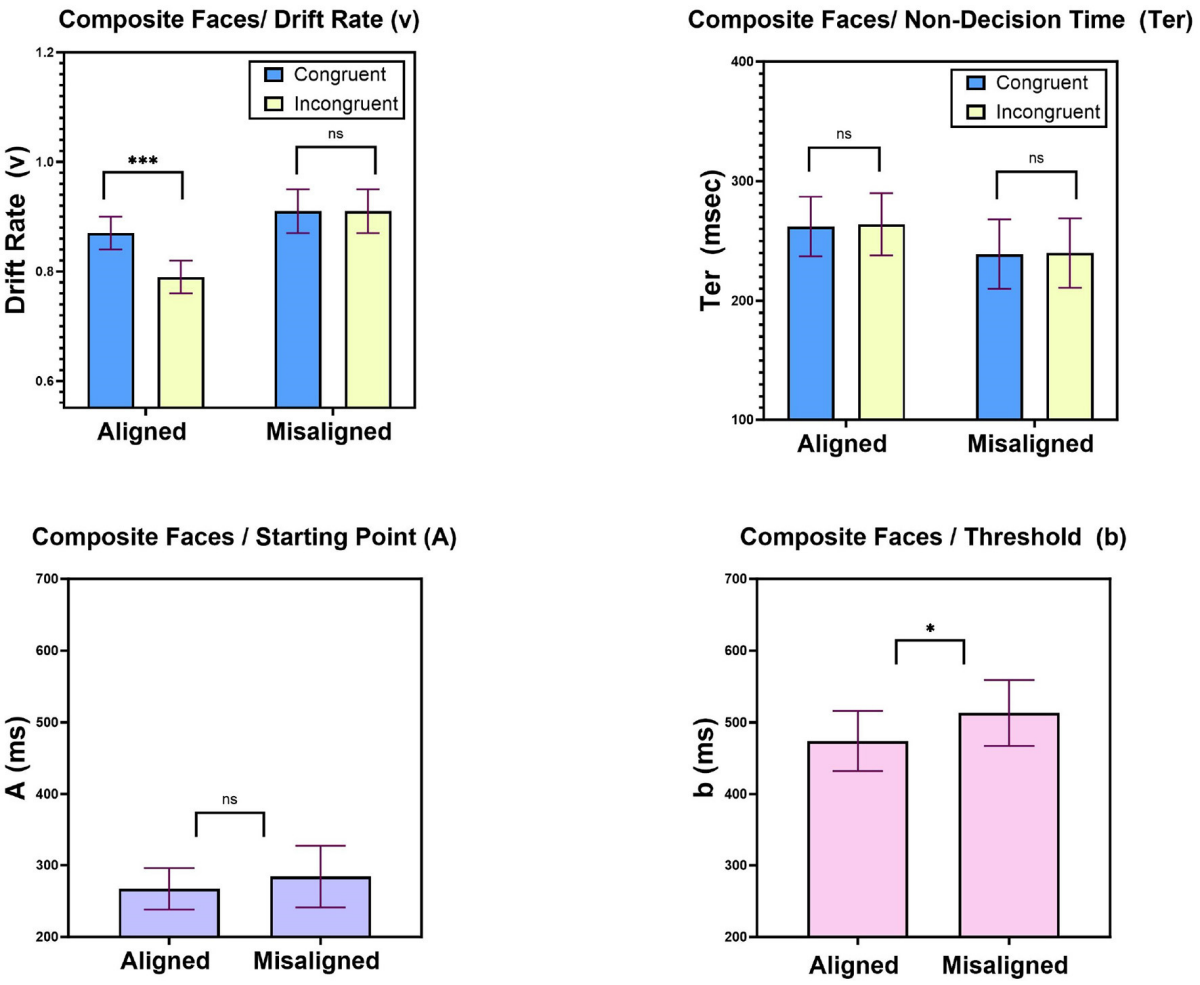
Experiment 2: mean LBA parameters. **Top left**: drift rates (v) as a function of Task (aligned, misaligned) and Congruity (congruent, incongruent), **top right**: non-decision times (Ter) as a function of Task (aligned, misaligned) and Congruity (congruent, incongruent). **Bottom left**: starting point (A) parameter as a function of Task (aligned, misaligned); **bottom right**: threshold (b) parameter as a function of Task (aligned, misaligned). Error bars are standard error of the mean. n.s, non significant; ^*^p < 0.05, ^**^p < 0.01, ^***^p < 0.001.

Figure 9 top left presents mean values of the drift parameter (v) as a function of Congruity (congruent, incongruent) and Task (aligned, misaligned). As can be noted, a congruity effect on drift-rates obtained in the aligned but not in the misaligned condition, such that in aligned condition the mean drift rate was higher in congruent than in in congruent trials. A two-way ANOVA with Task (aligned, misaligned) and Congruity (congruent, incongruent) supported this observation. A significant main effect of Task revealed higher drift rates in the misaligned (0.91) compared to the aligned (0.83) blocks [*F*_(1, 20)_ = 6.45, *MSE* = 0.12, *p* < 0.05]. A main effect of Congruity [*F*_(1, 20)_ = 13.3, *MSE* = 0.03, *p* < 0.01] showed a congruity effect that was modulated by Task [*F*_(1, 20)_ = 22.8, *MSE* = 0.032, *p* < 0.001], such that a congruity effect obtained in judgments of aligned composite faces [*F*_(1, 20)_ = 24.11, *MSE* = 0.06, *p* < 0.0001], but not in judgments of misaligned composites [*F* < 1]. These results entail that misaligned faces elicit more efficient accumulation of evidence, and most importantly, that changes in drift rate are a major contributing factor to the congruity effect observed in the aligned condition. These lead us to the conclusion that one of the psychological mechanisms that can account for the emergence of the composite face effect is related to the rate of evidence accumulation. We also found this mechanism to operate in the Navon figures, and this implies that at least in part, the two phenomena are affected by perceptual factors.

#### 9.2.2. Non-decision time

Figure 9 top right presents mean non-decision times as a function of Congruity and Task. A two-way ANOVA with Task (aligned, misaligned) and Congruity (congruent, incongruent) was performed on the best-fitting non-decision time (Ter) parameter values. No effect whatsoever was found to be significant (all *Fs* < 1). This suggests that neither Congruity nor Task had an impact on non-decision times. This is in contrast to the Navon figures, for which we recorded a significant influence. This is a major difference between the two phenomena.

#### 9.2.3. Starting point (A) and Threshold (b)

These parameters varied across Task (aligned, misaligned) while sharing their values for congruent and incongruent trials within a given task. The logic was that participants do not change their decision criteria within a block of trials, but only across blocks. To uncover their role in the composite face effect, we compared their best-fitting values between tasks using a two-sided paired *t*-test. Figure 9 bottom left, right, respectively, present the effects of Task (aligned, misaligned) on the LBA parameters of threshold (b) and starting point (b). With respect to starting point parameter (A), we did not record any significant difference between aligned (267 ms, SE = 29.5 ms) and misaligned (284 ms, SE = 43.8) conditions [*t*_(20)_ = −0.56, *p* = 0.57], whereas for threshold parameter (b), we did record a significant effect [*t*_(20)_ = −2.12, *p* < 0.05], such that lower threshold was observed in the aligned task (474 ms SE = 42.58) compared to the threshold in the misaligned task (513 ms, SE = 46.97). These results suggest that one of the psychological mechanisms responsible for the composite face effect is a change in the response threshold. It means that participants make more liberal decisions in the aligned condition. This result is opposite to that found with Navon figures, where the threshold parameter increased for the corresponding judgment of the local letter.

## 10. Discussion

Experiment 2 documented the composite face effect for both correct mean RTs and accuracy rates. The bottom-half interfered with judgments of the top-half for aligned composites, while this congruity effect was absent when classifying misaligned composites. Fitting LBA model parameters to RT distributions and accuracy rates from this experiment revealed that only two LBA parameters were involved in the generation of the composite face effect. First, drift rate was higher in congruent compared to incongruent trials, but only with aligned composites. This interaction mimicked the exact pattern observed with mean correct RTs and accuracy rates. Second, participants set a lower threshold with aligned compared to misaligned composites. The latter outcome is opposite to that observed with Navon figures where participant set a higher threshold in local-letter judgments. Taken together, the LBA results from Experiment 1 and Experiment 2, suggest that aside from the common influence of drift rates, Navon figures and composite faces are governed by different psychological mechanisms. Navon figures enhance changes in the non-decision times which reflect encoding and execution processes, whereas composite faces do not affect this parameter at all. In addition, with Navon figures participants raise the response threshold for judgments of the local letter, while with composite faces participant decrease the threshold for the analogous task of judging aligned composites. These findings cast serious doubts on the idea that Navon letters and composite faces are governed by the same psychological mechanisms. The two type of stimuli do exhibit similar mechanism of evidence accumulation, but with respect to all other LBA parameters they differ substantially.

## 11. General discussion

Navon compound letters (Navon, 1977; Miller and Navon, 2002) and composite faces (Young et al., 1987) serve as primary examples for hierarchical organization in perception. They have been often used to gauge holistic processes in object and face recognition. Many researchers believe that they capture the same species of holistic processing. As a result, the Navon task, along with the composite face task, have become routine procedures in testing prosopagnosic patients (Behrmann et al., 2005; Duchaine et al., 2007a,b; Avidan et al., 2011; Busigny and Rossion, 2011). The logic sustaining this joint application is that deficits in face perception are due to impaired holistic processing, and thus can occur with other non-face stimuli such as the Navon figures. The current investigation provides several lines of evidence against the view that Navon figures and composite faces are processed in a similar fashion. The evidence we adduced clearly shows that the surface similarity existing between the composite faces effect and Navon figures at the RT and accuracy level, conceals a more complex reality. The similarity between the two phenomena does not fully extend to the underlying psychological processes. First, at the level of mean correct and incorrect RTs, the composite face and the Navon effects exhibited different patterns. In the Navon task, incorrect RTs were found to be as fast as correct RTs, whereas in the composite face task, correct RTs were found to be faster than incorrect RTs. This is not a subtle difference. It attests to a profound difference in processing. The LBA accounts for relative speeds of correct and incorrect response times (Luce, 1986; Ratcliff and Rouder, 1998) through the interplay between start point and drift rate variability. In easy tasks, such as the Navon task, the response threshold *b* is fixed at or near the top of the start point distribution A. This leads to short integration times for both correct and incorrect responses. In difficult tasks, such as the composite face task, start point A is located far from the threshold *b*. As a result, integration time is longer, and correct response (with the higher drift rate) overtake the incorrect response (Brown and Heathcote, 2008).

A second line of evidence in favor of a dissociation between the composite face task and Navon's task comes from the opposite patterns of response threshold *b* setting. In the composite face task, participants set a higher response threshold for misaligned composites than for aligned composites. This criterion change is voluntary and implies that participants adopt a more conservative response in the misaligned condition. This also means that participants accumulate more evidence until they reach a decision with misaligned composite faces. The involvement of a criterion shift in the composite face task has been demonstrated by various researchers (Wegner and Ingvalson, 2002). However, in the Navon task, that pattern of threshold setting is exactly the opposite—participants set a lower response threshold in the global-letter task, which suggests a more liberal criterion placing.

A third line of evidence for the dissociation between the composite face task and Navon letter task comes from the non-decision time parameter *Ter*, which plays a role in the processing of Navon compound letters, but not in composite faces. Non decision times were found to be shorter in congruent than incongruent letters in the local-letter task, while this congruity effect was not present in judgment of the global letter. This outcome mimicked the Congruity × Task interaction obtained with correct RTs and accuracy. Non decision times reflect the duration of encoding and/or response execution stages. Our results entail that part of the Navon compound letters phenomenon is generated by these mechanisms, while in the composite face phenomenon these mechanisms play no role.

All of these suggest that despite a compelling surface similarity between the two tasks, Navon figures and composite faces may tap into separate psychological processes. This conclusion is also supported by recent findings from studies with prosopagnostic patients who demonstrated impaired composite face effect, but intact Navon effect (Duchaine et al., 2007b; Busigny and Rossion, 2011). The dissociation entails various theoretical implications for research on holistic or global processing.

A certain limitation of the current study should be highlighted. We tested two separate groups for the composite and Navon tasks to avoid carryover effects from one task to the other. However, this between-participant design does not allow us direct comparison between previously published and newly collected data.

Both Navon letters and composite faces are considered to be processed holistically. Our LBA results suggest that there might be various species of holistic processing mechanisms. Authors have long being arguing that the concept of holistic processing has various theoretical, operational, and measurement definitions (Garner and Morton, 1969; Maurer et al., 2002; Richler et al., 2012; Fitousi, 2013). Most of these definitions do not converge (Fitousi, 2015; Rezlescu et al., 2017). For example, when tested in Garner's speeded classification paradigm (Garner, 1974), top and bottom parts of composite faces appear as separable rather than integral dimensions (Pomerantz et al., 2003; Richler et al., 2013; Fitousi, 2015). Similarly, under matched discriminability, global and local dimensions in Navon figures, are found to be separable (non-interacting) (Pomerantz, 1983). It is apparent that existing definitions of what “holistic” might be are problematic. Some researchers have criticized these definitions for being poorly specified (Fitousi, 2015), and for thwarting progress in research on faces (Burton et al., 2015).

Another possibility is to assume that one of the tasks is not processed holistically in the first place. We Fitousi (2016, 2019, 2020a) and others (Cheng et al., 2018) have recently cast serious doubts on the idea that composite faces are processed holistically. The strongest evidence for this claim comes from a rigorous mathematical approach known as *system factorial technology* (SFT) (Townsend and Nozawa, 1995). Studies that applied the SFT to composite faces (Fitousi, 2015; Cheng et al., 2018) show that composite face parts are processed according to serial or parallel architectures, rather than the expected coactive architecture. This entails that face parts are not integrated into a genuine Gestalt, but continue to maintain their independent status. Moreover, when we have taken a closer look at the temporal dynamics of the composite face effect, we found the composite effect to increase in size over time (Fitousi, 2019; Lynch et al., 2020), in contrast to the expected global-to-local prediction (Navon, 1977; Busigny and Rossion, 2011). We conclude by asking researchers to exercise caution when administrating the Navon and composite face tasks in tandem. These two tasks and their associated stimuli cannot be put under the same umbrella, because they are processed differently.

## Data availability statement

The raw data supporting the conclusions of this article will be made available by the authors, without undue reservation.

## Ethics statement

The studies involving humans were approved by Ariel University Ethics Committee. The studies were conducted in accordance with the local legislation and institutional requirements. The participants provided their written informed consent to participate in this study. Written informed consent was obtained from the individual(s) for the publication of any potentially identifiable images or data included in this article.

## Author contributions

DF designed the experiments, analyzed the data, and wrote the paper. OA run the experiments. All authors contributed to the article and approved the submitted version.
